# Modular head-mounted cortical imaging device for chronic monitoring of intrinsic signals in mice

**DOI:** 10.1117/1.JBO.27.2.026501

**Published:** 2022-02-14

**Authors:** Mark Christian Guinto, Makito Haruta, Yuki Kurauchi, Taisuke Saigo, Kazuki Kurasawa, Sumika Ryu, Yasumi Ohta, Mamiko Kawahara, Hironari Takehara, Hiroyuki Tashiro, Kiyotaka Sasagawa, Hiroshi Katsuki, Jun Ohta

**Affiliations:** a Nara Institute of Science and Technology, Graduate School of Science and Technology, Division of Materials Science, Ikoma, Japan; b Kumamoto University, Graduate School of Pharmaceutical Sciences, Department of Chemico-Pharmacological Sciences, Kumamoto, Japan; c Kyushu University, Division of Medical Technology, Department of Health Sciences, Faculty of Medical Sciences, Fukuoka, Japan

**Keywords:** intrinsic optical signal, cortical imaging, CMOS, head-mounted devices, chronic imaging

## Abstract

**Significance:**

Intrinsic optical signals (IOS) generated in the cortical tissue as a result of various interacting metabolic processes are used extensively to elucidate the underlying mechanisms that govern neurovascular coupling. However, current IOS measurements still often rely on bulky, tabletop imaging systems, and there remains a dearth of studies in freely moving subjects. Lightweight, miniature head-mounted imaging devices provide unique opportunities for investigating cortical dynamics in small animals under a variety of naturalistic behavioral settings.

**Aim:**

The aim of this work was to monitor IOS in the somatosensory cortex of wild-type mice by developing a lightweight, biocompatible imaging device that readily lends itself to animal experiments in freely moving conditions.

**Approach:**

Herein we describe a method for realizing long-term IOS imaging in mice using a 0.54-g, compact, CMOS-based, head-mounted imager. The two-part module, consisting of a tethered sensor plate and a base plate, allows facile assembly prior to imaging sessions and disassembly when the sensor is not in use. LEDs integrated into the device were chosen to illuminate the cortical mantle at two different wavelengths in the visible regime ($\lambda_{\text{center}}$: 535 and 625 nm) for monitoring volume- and oxygenation state-dependent changes in the IOS, respectively. To test whether the system can detect robust cortical responses, we recorded sensory-evoked IOS from mechanical stimulation of the hindlimbs (HL) of anesthetized mice in both acute and long-term implantation conditions.

**Results:**

Cortical IOS recordings in the primary somatosensory cortex hindlimb receptive field (S1HL) of anesthetized mice under green and red LED illumination revealed robust, multiphasic profiles that were time-locked to the mechanical stimulation of the contralateral plantar hindpaw. Similar intrinsic signal profiles observed in S1HL at 40 days postimplantation demonstrated the viability of the approach for long-term imaging. Immunohistochemical analysis showed that the brain tissue did not exhibit appreciable immune response due to the device implantation and operation. A proof-of-principle imaging session in a freely behaving mouse showed minimal locomotor impediment for the animal and also enabled estimation of blood flow speed.

**Conclusions:**

We demonstrate the utility of a miniature cortical imaging device for monitoring IOS and related hemodynamic processes in both anesthetized and freely moving mice, cueing potential for applications to some neuroscientific studies of sensation and naturalistic behavior.

## 1 Introduction

Optical imaging methods have been instrumental in deciphering the functional roles of numerous brain structures in live animals across different contexts, providing insights into the mechanistic, physiological, and metabolic bases of neurological disease progression and aging.^1^^–^^3^ Likewise, the ability to directly visualize changes in brain activity has facilitated mapping^4^^,^^5^ and remapping^6^^–^^8^ investigations of the cortex at mesoscopic scales. Although electrocorticography arrays are prone to damaging tissue as probes come into contact with or are inserted beneath the dura, optical imaging allows for a less invasive means of monitoring cortical dynamics relating to neurons (and possibly glia), the vasculature, and their interactions.^9^^,^^10^ In practice, such cortical dynamic correlates can be visualized by tracking local blood flow velocity (hemodynamics)^11^ or by detecting intrinsic optical signals (IOS) that are endogenously generated in the brain.^12^^,^^13^ IOS imaged from the cortical mantle of an intact brain, particularly in the somatosensory cortex, have been shown to be associated with physiological responses of the animal during periods of sensory stimulation.^14^ Multiple lines of evidence suggest that these endogenous responses, which are often weaker in intensity than those observed in extrinsic fluorescence studies, arise from dynamic metabolic processes in the brain, including hyperemia or the local increase in blood flow, cytochrome-related activity, and ${\mathrm{NAD}}^{+}/\mathrm{NADH}$ redox transformation.^15^^–^^17^ Further, differential reflectance measurements on the brain surface indicate various metabolic/physiological states that different cortical domains may assume, appearing lighter or darker for a short period relative to a prestimulus baseline.^18^^,^^19^

At present, while head-mountable modules^20^^–^^29^ exist for monitoring functional changes in cortical activity in rodents, several of which support studies in freely behaving conditions, these systems have so far precluded tracking long-term mesoscopic cortical dynamics in mice because the devices themselves are too heavy to be mouse compatible (i.e., they are mostly suitable for rats). Multimodal head-mountable microscopes built to accommodate hemodynamic imaging^20^^,^^26^^–^^29^—which include IOS imaging and cerebral blood flow tracking via laser speckle contrast analysis—have weights ranging from 3 to 20 g and are typically 5 cm^3^ in size, mainly due to the inclusion of lenses, fiber bundles, and/or laser diodes in their design in order to approximate the resolving performance of benchtop imagers. Lightweight imaging systems that are particularly suited for studies in mice are nevertheless sought after because of the wider array of genetic tools and extensive neuroanatomical resources available for mice.^4^^,^^30^ A miniature, head-mounted, wide-field imaging device that is sufficiently light for use in mice experiments is expected to open avenues for investigating cortical dynamics under a variety of naturalistic, freely moving behavioral settings.

Indeed, in calcium imaging studies, fluorescence microendoscopes^30^^–^^37^ have been optimized for monitoring neuronal ensemble activity in various small animals expressing genetically encoded calcium indicators (GECIs).^38^^,^^39^ State-of-the-art models that satisfy different imaging requirements include nVista (pioneering epifluorescence microscope, weighing 1.8 g and up depending on the unit),^31^ UCLA miniScope V4 (pioneering open-source platform; high-resolution achromatic optics and automatic focus, 2.6 g),^36^ NINscope (multiregion imaging, 1.6 g),^34^ wireless miniScope (untethered, 4.5 g),^32^^,^^35^ and FinchScope (designed for zebrafinch investigations, 1.8 g).^37^ These devices show promise and continue to bring mechanistic insights into both functional and dysfunctional activity within various superficial, subcortical, and deep brain regions, including, but not limited to the cortex,^31^ subfields of the hippocampus,^32^^,^^33^ the cerebellum,^34^ and the striatum.^35^ Although a baseplate, headplate, or chronic cranial window component is also present in the design of these devices, similarly enabling longitudinal imaging of brain activity, our approach employs apposable fiber-optic plates (FOPs) that are suitable for contact imaging. The dispensability of lens components in this setup maintains the light weight of the head-mountable device.

In this paper, we propose a method for realizing long-term observation of IOS in mice at two illumination wavelengths using a compact imaging device that readily allows freely moving experiments to be implemented. To our knowledge, the device presented herein for monitoring intrinsic signals offers the lightest weight and smallest form factor. Previous studies have already demonstrated the utility of CMOS image sensor-based imaging devices in probing brain function both in the cortical surface^40^^,^^41^ and in the deep brain.^42^^,^^43^ However, since the device packaging has not been optimized for longitudinal studies, issues such as blurring of acquired images, device breakage, and stress inflicted on animals have been encountered.

To address these, we have developed a modular device that can be implanted for a long period of time in mice without any perceptible degradation in imaging performance. The imaging device is composed of two parts: (1) a sensor plate which houses the CMOS image sensor chip and LED illumination sources; and (2) a permanent head plate which is affixed to the skull of the mouse and serves as a cranial window. Integrating FOPs into the modular design enabled facile assembly of the fully functioning device prior to the recording session, obviating the need for elaborate setup preparations. FOPs also serve to protect the image sensor and the LEDs and allow the sensor plate to be reusable across multiple experiments. In addition, minimization of the number of pads in the image sensor design has afforded a reduced form factor for the device. The incorporation of the image sensor, the LEDs, and the FOPs to the jig frames—a pair of precision-milled plates that ensure snug fit and accurate alignment of the FOPs—has resulted in a cortical imaging device that, in total, weighs only 0.54 g. The light weight of this device poses minimal interference to freely behaving small animals like mice and is expected to suit *in vivo* experiments requiring awake, unrestrained conditions.

In the following sections, the device design, fabrication process, and IOS data analysis will be described in more detail. Evaluation of the device will be carried out by monitoring the hindlimb (HL) receptive field of the somatosensory cortex in response to cutaneous stimuli and by observation of mouse behavior in freely moving conditions. Furthermore, estimation of blood flow speed from the cortical imaging session in a freely behaving mouse will be demonstrated as a proof of the utility of the device.

## 2 Materials and Methods

### 2.1 Overview of the Modular Head-Mounted Cortical Imaging Device

**Fig. 1:**
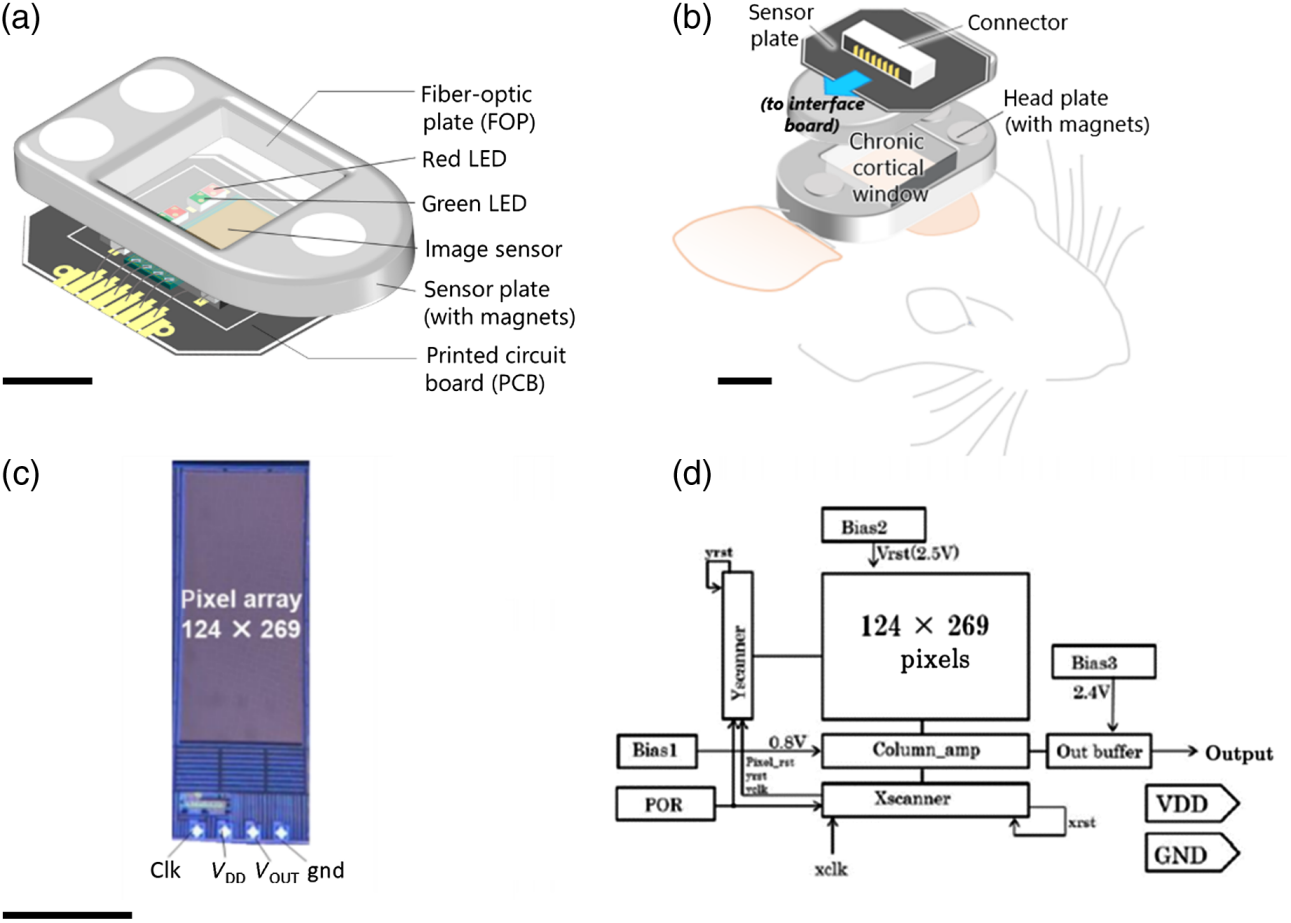
Schematic of the modular head-mounted imaging device for monitoring IOS in rodents. (a) Details of the sensor plate containing the CMOS image sensor and LEDs ($\lambda_{\text{center},\text{green}}=535\;\mathrm{nm}$ and $\lambda_{\text{center},\text{red}}=625\;\mathrm{nm}$) embedded on a PCB. A black ink used for light shielding was not drawn for clarity. Scale bar: 2 mm. (b) Assembly of the sensor plate (shown turned over) and the head plate as implanted on the mouse prior to imaging. Both plates are fitted with FOPs that serve as protective covering and as a window into the exposed cortex. Wires connecting the sensor plate to the interface board are omitted for simplicity. Scale bar: 2 mm. (c) Photograph of the CMOS image sensor chip (124  px×268  px) with the four pads indicated: clock (clk), $V_{\mathrm{DD}}$, output ($V_{\text{OUT}}$), and ground (gnd). Scale bar: 1 mm. (d) Block diagram of the CMOS sensor circuit.

As mentioned in Sec. 1, a two-part modular design for the imaging device comprising a sensor plate and a permanent head plate was developed. The device is capable of imaging the cortical surface including the superficial vasculature. As shown in Fig. 1(a), the image sensor is flanked by LEDs [ES-CEGHA14A, $\lambda_{\text{center}}=535\;\mathrm{nm}$ (green), $\Delta \lambda_{\text{half-width}}=35\;\mathrm{nm}$ (at forward current $I_{f}=20\;\mathrm{mA}$), 300  μm×300  μm; ES-AEHRAX12, $\lambda_{\text{center}}=625\;\mathrm{nm}$ (red), $\Delta \lambda_{\text{half-width}}=18\;\mathrm{nm}$ [(at forward current $I_{f}=20\;\mathrm{mA}$)], 300  μm×300  μm Epistar Corp., Taiwan] of two different center wavelengths intended for monitoring IOS that predominantly involve changes in blood volume (volumetric) and oxygenation states of hemoglobin (oximetric), respectively.^19^ Six LEDs for each color were distributed in an alternating pattern and spaced evenly around the image sensor to maximize illumination coverage. An aluminum jig designed to fit an FOP is placed on top of the sensor and LEDs. Three small Nd magnets were embedded into the jig, allowing the sensor plate to neatly attach onto the head plate which is also equipped with magnets [Fig. 1(b)]. The head plate, which can be considered as a chronic window module, serves as physical protection and allows optical imaging of the exposed cortex after attachment to the sensor plate (imaging module). The dedicated CMOS image sensor has dimensions of 1048  μm×2700  μm×250  μm, with the pixel array occupying a sizeable area of the chip (900  μm×1920  μm). Both the chip and the LEDs are mounted to a custom printed circuit board (PCB), in which the anodes and cathodes of the LEDs, the clock signal (clk), $V_{\mathrm{DD}}$, output signal ($V_{\text{out}}$), and ground (gnd) pads of the chip are connected [Fig. 1(c)]. The block diagram of the CMOS circuit is shown in Fig. 1(d) where the nominal voltages of the multiple biases are specified. Table 1 provides the specifications of the customized image sensor. The image sensor was designed and fabricated using a standard CMOS process (0.35-μm, 2-poly 4 metal CMOS; Taiwan Semiconductor Manufacturing Co., Ltd., Taiwan). The chip holds 124×268  pixels and each pixel has a size of 7.5  μm×7.5  μm.

**Table 1:** Specifications of the CMOS image sensor chip.

Technology	0.35-μm 2-poly 4-metal standard CMOS process
Operating voltage	3.3 V
Chip size	1048 μm×2700 μm
Pixel array size	900 μm×1920 μm
Pixel type	Three-transistor active pixel sensor
Photodiode	n-well/p-sub
Pixel size	7.5 μm×7.5 μm
Pixel count	33,232
Fill factor	44%

Since head-mounted imaging devices in live subjects, regardless of their size, demand small form factors and minimal invasiveness, the use of a customized image sensor with only four I/O pads is particularly advantageous. Keeping the number of wires between the imaging sensor chip and the external board at a minimum has reduced the size and weight of the sensor plate. In this regard, unlike commercially available CMOS image sensors, our image sensor is not equipped with a general on-chip analog-to-digital (A/D) converter so that A/D conversion is performed off-chip. The imaging system consists of a control board, in which an A/D converter and voltage regulators are mounted; a relay board that transmits signals between the imaging device and the control board; and a personal computer (PC). The imaging device is controlled by a PC through these boards, and the captured image is recorded and visualized by a custom control program written in C++. In addition, the control program can display differences between images against a reference in real time and is configured to facilitate fine parameter tuning during *in vivo* experiments.

By taking the contact imaging approach in which the device is directly placed against the tissue of interest, bulky lenses were eliminated and further miniaturization was achieved. Furthermore, the use of FOPs as an integral part of the cranial window enables contact imaging and, at the same time, serves to protect the surface of the brain. FOPs have a structure consisting of an array of optical fibers (Ø3.0  μm), allowing them to effectively translate imaging planes across the fiber axis.^41^^,^^44^ Exact alignment of the FOP fiber bundle and CMOS sensor pixel array was not found to be critical to the imaging quality since the pixel pitch of the CMOS image sensor (7.5  μm) is more than twice the size of the individual fibers of the FOP. The sensor plate, on average, weighs 0.34 g, whereas the head plate weighs 0.20 g. Overall, the average weight of the modular device is 0.54 g and its size is 8.2  mm (L)×6.4 mm (W)×7.2  mm (H).

### 2.2 Device Fabrication

In designing a mounting PCB for the image sensor and LEDs, our primary aim was to limit its size so that it does not occupy an area larger than the aluminum jig. This is to avoid uncoupling of the sensor plate during *in vivo* imaging experiments. Four I/O pads of the mounting board were dedicated to the CMOS image sensor clk, $V_{\mathrm{DD}}$, $V_{\text{OUT}}$, and gnd lines and the remaining four I/O pads were intended for the terminals of the arrays of green and red LEDs. LEDs located on the same flank share the same ground line. I/O pads were bonded to Al wires (Ø25  μm) using a wedge bonder (7700CP, West Bond Inc., USA). In addition, jigs were crafted to facilitate long-term implantation. These jigs allow the plates to be mounted neatly into each other without impeding the movement of the mouse. For this purpose, aluminum was deemed to be a suitable material because it is lightweight and can be easily machined on the order of microns. Both jigs for the sensor and head plates have rectangular windows through which 0.5-mm-thick FOPs (J5734-Y029, Hamamatsu Photonics, Japan) were fitted; furthermore, holes (Ø1.5  mm) were made in the jig where the Nd magnets (D101-N5, K&J Magnetics, Inc., Pennsylvania, USA) could be embedded. A thermosetting epoxy resin (Z-1 Nissin Resin, Japan) was used to fix the FOPs and magnets in place. The FOP window spans 3.0  mm×4.0  mm, ensuring full coverage of the pixel array area of the CMOS image sensor and the surrounding LEDs. A black ink (Canon Chemicals, Inc., Japan) was coated around the periphery of the device to block incoming stray light. An 8-pole crimp connector pad was then soldered on the back side of the mounting board. This configuration allowed the sensor plate to easily interface with the control board.

Clock input and output signals from the image sensor, as well as the supply power, were transmitted via shielded wires (#28AWG ultra-fine 6-core shielded cable, MOGAMI, Japan) in a twisted pair configuration, protecting the signal lines from noise pickup by minimizing electromagnetic interference. Furthermore, we have incorporated a relay board to the cable for noise suppression (Fig. S1 in the Supplementary Material). This intermediate component was equipped with a digital buffer circuit on the input side and a unity-gain operational amplifier that achieves high slew rates and low noise on the output side. In addition, a noise filter circuit was included to stabilize the power supply line. In contrast to a previous design, which had a large form factor and therefore had to be placed on the backs of mice and rats,^40^ the current relay board has a compact rod shape so that it can conveniently hover above the head of the mouse. It is expected that this will further reduce the stress induced on the animal during *in vivo* behavioral experiments.

### 2.3 Stereotaxic Surgery

Mature wild-type mice (C57BL/6, Japan SLC Co., Ltd., 8 to 10 weeks old) were used in this study. All animal experiments were approved by the Animal Care and Handling Committee of the Nara Institute of Science and Technology. For tactile stimulation experiments in restrained mice, urethane (ethyl carbamate; 10% w/v, Wako Pure Chemical Industries, Inc., Japan) was administered intraperitoneally as systemic anesthesia at a weight-dependent dose (1  mL/kg). By contrast, for the freely moving session, surgical implantation of the head plate was performed using sodium pentobarbital (Kyoritsu Seiyaku Corp., Japan) administered intraperitoneally (50 mg/kg) to promote subsequent recovery of the mouse. After the anesthesia has induced loss of consciousness and sensation, the scalp hair of the mouse was trimmed and it was set to a stereotaxic apparatus (SR-5M, Narishige Scientific Instruments, Japan), ensuring that its head was held securely in place by means of an ear bar and a nose clamp. Xylocaine (2%, 20  mg/mL, AstraZeneca, UK) was applied topically around the scalp of the mouse. Heating pads were used to maintain the body temperature within a safe range throughout the operation. Incision of the scalp and subsequent removal of the subcutaneous membrane revealed the cranial surface. Chlorhexidine gluconate (0.05%, Sumitomo Dainippon Pharma Co., Ltd., Japan) was applied as a general antiseptic. Physiological saline was regularly irrigated to the exposed surgical site to prevent dehydration. To access the primary somatosensory cortex-forelimb/hindlimb receptive fields (S1HL/FL), a 2  mm×3  mm-sized bone flap (centroid: –AP: 0.5 mm, ML: +1.5  mm) was removed by drilling. Dura mater was left intact. The basal surface of the FOP (head plate) was carefully placed in contact with the dura overlying S1HL/FL. A small metal weight was placed on top of the head plate to induce sufficient pressure on the brain surface, minimizing the risk of bleeding and ensuring that the entire exposed tissue is covered.^45^ Stainless steel self-tapping screws were subsequently affixed to the cranium to facilitate adhesion of the dental cement (Super-Bond, Sun Medical Co., Ltd., Japan) that was applied at the end of the surgical operation.

### 2.4 Mechanical Stimulation Sequence

**Fig. 2:**
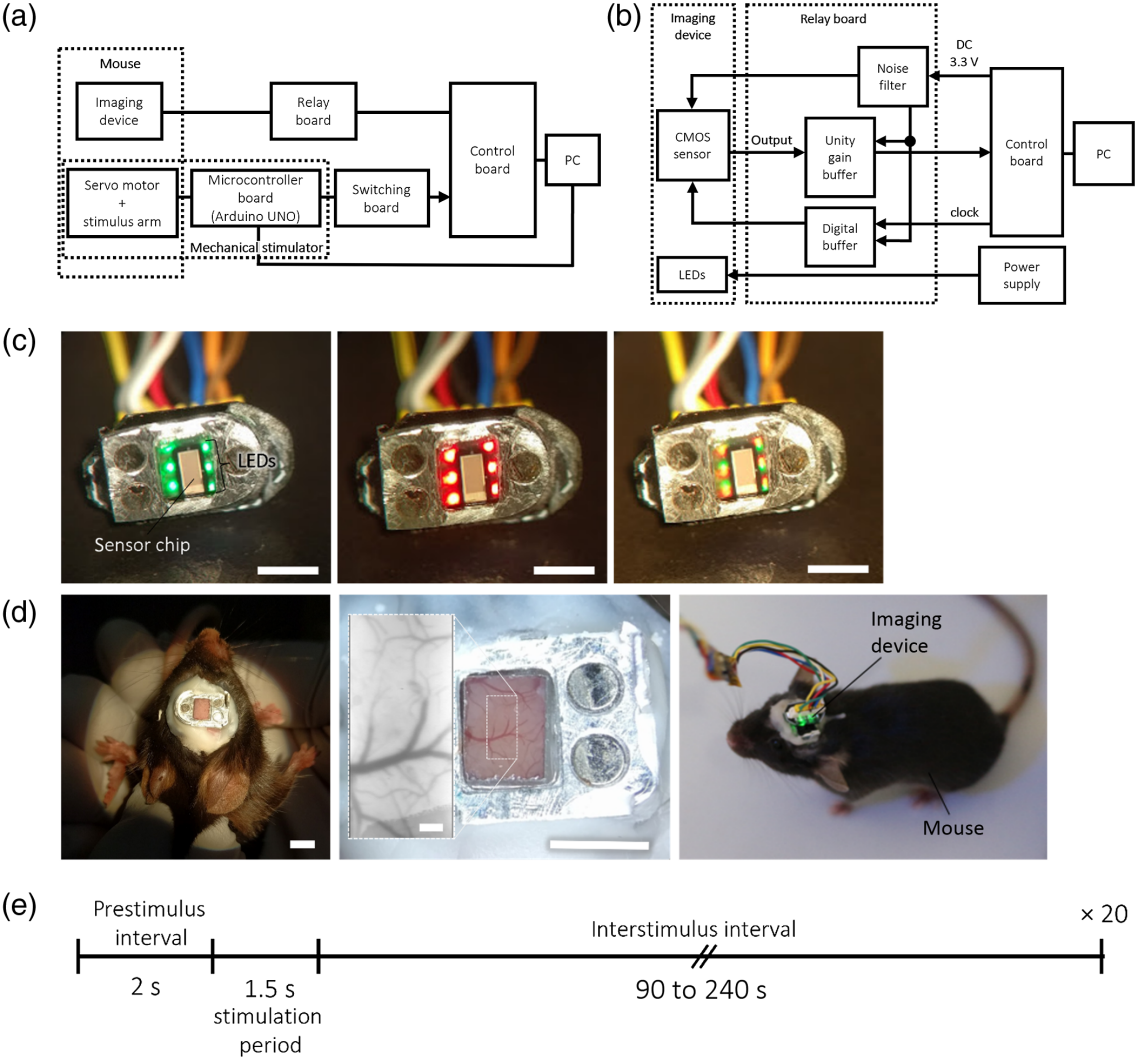
IOS imaging in mice using the modular head-mounted device. (a) Block diagram of the combined mechanical stimulation and imaging system. The tact switch is integrated to the microcontroller board. (b) Block diagram of the relay board, consisting of buffers and a noise filter, and the imaging device. (c) Photographs of the sensor plate indicating the placement of the image sensor chip and the LEDs. From left to right: device operating with green LEDs, red LEDs, and both types of LEDs. Scale bar: 3 mm. (d) Left: restrained wild-type C57BL/6 mouse implanted with a head plate, allowing optical access into the left cortical hemisphere. Scale bar: 3 mm. Middle: close-up photograph of a head plate 40-days postimplantation, fixed onto the cranium with dental acrylic. Cortical surface vasculature is clearly visible underneath the FOP. A single unbinned frame of the imaged area (dotted rectangle) as captured by the image sensor is shown as inset. Scale bar: 3 mm (head plate); 200  μm (image sensor, inset). Right: the imaging device, consisting of the sensor plate and head plate, secured on the head of a mouse. (e) Stimulation sequence for the plantar surface of the hindpaw of an anesthetized mouse. The interstimulus interval varies from 90 to 240 s. Each stimulus condition (contralateral or ipsilateral) lasted for 20 consecutive trials.

In observing IOS, it was deemed necessary to perform stimulation of the HL multiple times due to the relatively weak amplitude of these endogenously generated signals. Averaging across trials mitigates artifactual contributions related to heartbeat and respiration.^46^ Since the onset of stimulation needs to be tracked and annotated with respect to the timing of the intrinsic signal responses, an instrument was created to periodically stimulate the HL of mice and transmit a trigger signal to the imaging system. The schematic of the combined stimulation and imaging systems is illustrated in Fig. 2(a). The stimulator consists of a servo motor (SEEED-316010005, Switch Science, Japan), a stimulus arm (a blunt needle), a control microcomputer board (Arduino UNO), an LCD monitor (SEEED-104020112), a tact switch, and a stimulus setting PC. The control microcomputer board controls the servo motor, sends trigger signals, and receives stimulus settings. The I/O signals of the sensor were transmitted from the wiring to the relay board, as shown in Fig. 2(b), which was then connected to the control board. Analog signals from the control board were converted digitally and acquired by the PC through an interface card. Signal processing and setting controls were performed by the control program. In addition, a switching board that operates in response to the trigger signal from the microcomputer board was inserted between the stimulator and the control board. The spatial resolution of the assembled device was found to be ∼22.1  μm/line-pair (45.3  lp/mm) through contact imaging of the 1951 USAF resolution test target, which is sufficient for differentiating macrovascular and parenchymal signals.^47^ In this experiment, imaging was performed at a frame rate of 133 fps.

IOS were then recorded from the S1HL/FL receptive fields and surrounding areas during stimulation of the contralateral and ipsilateral HL of urethane-anesthetized, head-restrained mice (N=5). Green and red LEDs were used to illuminate the brain surface and monitor, chiefly, blood volume, and oxygenation state-related changes in IOS, respectively. Photographs of the sensor plate as shown in Fig. 2(c) indicate the placement of the image sensor chip and the LEDs under operation. Figure 2(d) shows the head plate implanted on the mouse. The cranial window clearly shows the cortical surface and its vasculature underneath the FOP, in which a $1.72\text{-}{\mathrm{mm}}^{2}$ area can be imaged by the sensor chip. The assembled imaging device, comprising the sensor and head plates, can be secured quickly on the head of a mouse in both anesthetized and awake conditions. After an initial recording that lasts for 30 s, HL stimulation was delivered for 1.5 s when the swinging arm pressed upon the plantar surface of the hindpaw at a constant pressure. The last 2 s immediately preceding the stimulus onset was taken as the prestimulus interval [Fig. 2(e)]. A random interstimulus interval was set between 90 to 240 s, after which the stimulation was delivered again, marking the next trial. Four 20-trial recordings were taken in each mouse, corresponding to each stimulation condition: contralateral (green and red illumination) and ipsilateral (green and red illumination). The order of the stimulation condition was randomized across different mice. IOS recordings for each color of illumination (green or red LEDs) were obtained from separate but contiguous sessions. Imaging sessions were carried out either immediately after the head plate implantation on the mouse or after a 40-day recovery period.

### 2.5 Data Processing

Imaging data were obtained using a customized program for the imaging system (CIS_NAIST, C++) and processed in MATLAB (R2020a, MathWorks, Natick, Massachusetts, USA). Each recording was scanned for stimulus delivery periods (1.5 s, 20 instances) and the first frame that indicated the onset of stimulus for each trial was set to time zero (t=0). Then 400 frames (∼3  s) prior to and 2500 frames (∼19  s) following the stimulus onset for every trial were collected. The fractional change in intensity ($\Delta I/I_{0}$) of each frame relative to the mean of all 3000 frames per trial was computed to create a new array of monochromatic frames. Trial-averaging, normalization, and first-difference time series detrending were applied for each stimulation condition to obtain a 124×268×3000 array. Five regions of interest (ROIs) having an area of 20  px×20  px were selected among the parenchymal domains (areas devoid of large surface blood vessels) within the field of view and were used to construct line plots. Areas that subsume superficial blood vessels were excluded from the IOS analysis since both vascular flow and heterogeneous composition of blood are known to result in aberrant changes in signal reflectance.^48^ Temporal filtering (moving average; window length = 20 frames) was applied to smoothen out the plots. Activity maps were created from averaging 200 consecutive frames (1.5-s bins), revealing multiphasic IOS profiles across a 22-s epoch. Cursory estimates of the relative contrast between the response maps were made using the histogram function of the Fiji distribution of ImageJ (National Institutes of Health, Bethesda, Maryland, USA). The standard deviation (SD) of the frequency distribution of grayscale values was used as a metric^49^ which reflected the degree of difference between the darkest and brightest parts of a particular set of response maps.

To demonstrate the capability of the imaging device in obtaining blood flow speed estimates, cortical recordings under green LED illumination were taken from a freely behaving mouse. Video playback (AVI format) of cortical recordings was created using MATLAB. Line ROIs were specified using Fiji to construct kymographs. A high SNR reference image used for tracing blood vessels was obtained by temporal averaging in 7.5-s bins (1000 frames), resulting in an artifactual lightening of the blood vessels relative to the parenchyme. Blood flow speed ν across a specific time interval was estimated using the following equation:

ν=F·ΔP·tan θ, (1)

where F is the mean frame rate of the image recording, ΔP is the pixel pitch, and θ is the angle formed by the striations in the kymograph relative to the spatial axis.

### 2.6 Immunohistochemistry

Immunohistochemical analysis was performed to evaluate cortical tissue response to the implanted device after a recording session. Although the dura was ensured to remain intact during cortical window preparation (mice surgeries that resulted in punctured dura or abnormal bleeding rates were excluded from the IOS recording experiments), verifying the safety of the device operation was still necessary since LED illumination may induce a phototoxic effect on the tissue. Adverse immune response in the cortex can be checked using glial markers; the glial distribution in the imaged hemisphere can be compared with that of the opposite, nonimaged hemisphere to assess for abnormalities.^50^ Cumulatively, the green and red LEDs were turned on for at least 2 h to obtain the IOS recordings. Prior to brain extraction, mice were deeply anesthetized in sodium pentobarbital (200  mg/kg; Kyoritsu Seiyaku Corp., Japan) and transcardially perfused with 4% paraformaldehyde solution in PBS (PFA; Wako Pure Chemical Industries, Inc., Japan). Euthanasia and, subsequently, perfusion fixation were performed immediately after the imaging session was finished. Fixed brains were extracted and equilibrated in 4% PFA for 1 h and transferred to cold PBS mixed with 0.1% sodium azide (Wako Pure Chemical Industries, Inc., Japan). Coronal sections (40-μm thickness) were prepared using a vibratome (Linear Slicer Pro7, Dosaka Em Co. Ltd., Japan). Blocking and permeabilization treatment in 5% bovine serum albumin and 0.5% Triton X in PBS was done for 1 h. Subsequently, slices were incubated at 4°C overnight with the primary antibody diluted in PBS. Slices were then reincubated with the secondary antibody in PBS at room temperature for 1 h and mounted on glass slides in an antifade mounting medium (Vectashield, Vector Labs, Burlingame, California, USA). The following antibodies were used for staining: anti-NeuN antibody, clone A60 (1:1000; MAB377, Millipore), anti-CD68 antibody (1:200; ab53444 rat, Abcam), and anti-GFAP antibody (1:1000; ab7260 rabbit, Abcam), goat anti-mouse IgG (1:800; H + L, A-21208, Alexa Fluor 488), donkey anti-rat IgG (1:800; H + L, A-21208, Alexa Fluor 488), and goat anti-rabbit IgG (1:800; H + L, A-11011, Alexa Fluor 568). Imaging of the tissue was done using an inverted microscope (DMI6000, Leica Microsystems, Germany) with a 10× objective. Image analysis was performed using Fiji. Cell count (neurons, astrocytes, and microglia) as a function of cortical depth at 50-μm increments was aided by a standardized sequence of contrast enhancement, thresholding, and “analyze particles” functions. Statistical significance was assessed using a two-tailed Mann–Whitney U test (α=0.05).

## 3 Results

### 3.1 Intrinsic Optical Signals in Response to Hindlimb Stimulation

**Fig. 3:**
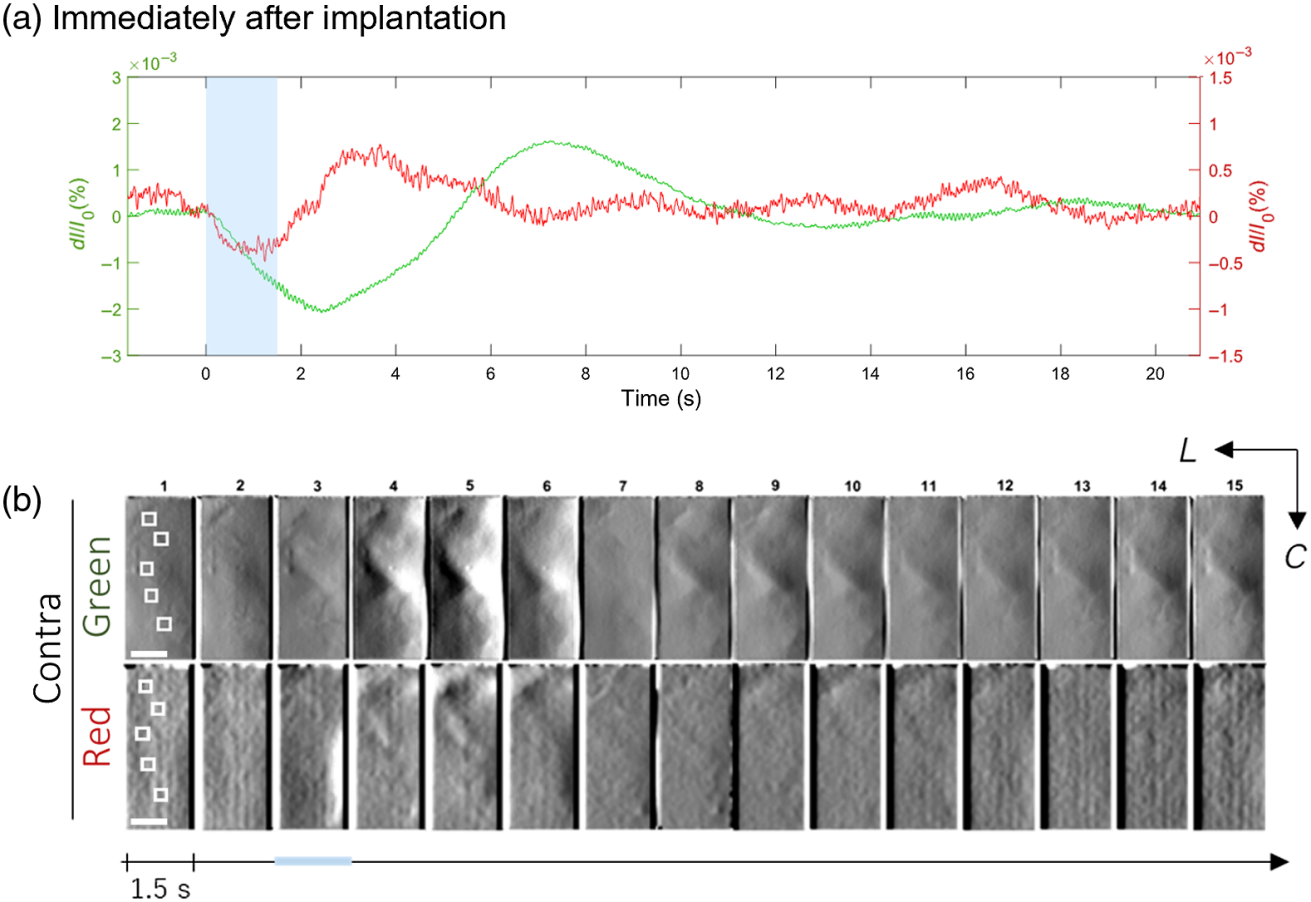
Stimulus-evoked IOS in the HL receptive field (S1HL) of anesthetized wild-type mice, recorded immediately after implantation. (a) Representative line plots of the fractional change in intensity values ($\Delta I/I_{0}$) averaged from five ROIs (20  px×20  px, slightly enlarged in the image for clarity) within the cortical recordings (N=5) and across 20 trials. IOS obtained under red and green illumination modes are plotted on the same graphs; scaling of the y axes was adjusted to clearly distinguish signal peaks. Contralateral stimulation (delivery period indicated by the blue band, 1.5 s) elicited differential IOS profiles. ROIs were selected to predominantly include the parenchyme and exclude large surface vessels. (b) Activity maps constructed from 1.5-s bins of trial-averaged acute cortical recording, revealing darkening and brightening of areas that characterize the phases of IOS profiles. The imaging area is 900  μm×1920  μm. Scale bar: 500  μm. Lookup table (LUT): $-2.3\times 10^{-3}$ to $1.5\times 10^{-3}$ $\Delta I/I_{0}$ (green); $-0.5\times 10^{-3}$ to $0.5\times 10^{-3}$ $\Delta I/I_{0}$ (red). Contralateral HL responses were recorded under green and red LED illumination. Stereotaxic directions are specified L (lateral) and C (caudal). As with (a), stimulation delivery period is indicated by the blue bar that lasts 1.5 s.

Representative line plots of the stimulus-evoked IOS in S1HL of anesthetized wild-type mice are shown in Fig. 3(a). Trial-averaged responses were collected under both green and red LED illumination. The stimulus delivery period lasted 1.5 s as indicated by the blue band. From the ROIs, a triphasic profile exhibited by the IOS under green illumination and contralateral stimulation is found, whereby the fractional change in intensity drops almost immediately upon HL stimulation and lasts for around 5 s (and the trough is reached halfway at ∼2.5  s), after which the signal intensity rises and lasts at around 11 s (peak: ∼7  s). An accompanying undershoot is then observed between 11 and 14 s (duration: ∼3  s; trough: ∼13  s). Interestingly, supplemental line plots of individual trials across three mice for contralateral-green illumination display consistent initial dips that are time-locked to the stimulation onset (Fig. S2 in the Supplementary Material), with the exception of the first trials. The imaging session was carried out immediately after the head plate implantation on the mouse.

For red illumination, a similar triphasic profile can be observed but at a more compressed timescale: the initial dip lasts around 2 s (trough: ∼1  s), the overshoot around 4 s (peak: 3.5 s), and the undershoot around 4 s (trough: ∼7  s). For ipsilateral stimulation, a smaller amplitude response can be detected from the green illumination and is undetectable for the red stimulation (Fig. S3 in the Supplementary Material). However, distinguishable peaks appearing from between 15 and 18 s for both green and red illuminations suggest either a delay or recurrence of signals that may propagate from the contralateral side to the ipsilateral side of the brain. Notably, in a voltage-sensitive dye imaging study by Ferezou et al.,^11^ it had also been demonstrated that the ipsilateral hemisphere generates weak but significant, long-latency responses following whisker stimulation.

Furthermore, in Fig. 3(b), activity maps were created from 1.5-s bins of a trial-averaged acute cortical recording. Lateral (L) and caudal (C) stereotaxic directions are specified. Darkening and brightening of areas that characterize the phases of IOS profiles are apparent especially in the contralateral-green illumination condition where signal amplitudes can clearly be grouped into three phases. On the other hand, the lower contrast between images for the contralateral-red illumination are reflected from the weaker amplitudes observed in Fig. 3(a). For both ipsilateral stimulation under both red and green illumination, a significant IOS response (taken to be >0.5 SD, from mean of distribution) at the onset of mechanical stimulation was not observed; this, too, is reflected in the relatively weak amplitudes of the IOS signals that propagate after some delay in the ipsilateral hemisphere (Fig. S3 in the Supplementary Material).

**Fig. 4:**
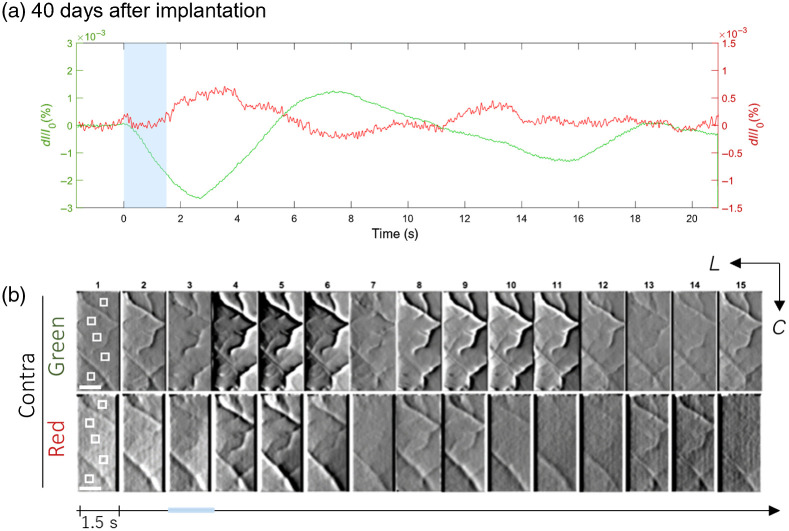
Stimulus-evoked IOS in the HL receptive field (S1HL) of anesthetized wild-type mice, recorded 40 days postimplantation. (a) Representative line plots of the fractional change in intensity values ($\Delta I/I_{0}$) averaged from five ROIs (20  px×20  px, slightly enlarged in the image for clarity) within the cortical recordings and across 20 trials. IOS obtained under red and green illumination modes are plotted on the same graphs; scaling of the y axes was adjusted to clearly distinguish signal peaks. Contralateral stimulation (delivery period indicated by the blue band, 1.5 s) elicited differential IOS profiles. ROIs were selected to predominantly include the parenchyme and exclude large surface vessels. (b) Activity maps constructed from 1.5-s bins of trial-averaged acute cortical recording, revealing darkening and brightening of areas that characterize the phases of IOS profiles. The imaging area is 900  μm×1920  μm. Scale bar: 500  μm. Contralateral HL responses were recorded under green and red LED illumination. Stereotaxic directions are specified L (lateral) and C (caudal). LUT: $-2.3\times 10^{-3}$ to $1.5\times 10^{-3}$ $\Delta I/I_{0}$ (green); $-0.5\times 10^{-3}$ to $0.5\times 10^{-3}$ $\Delta I/I_{0}$ (red). As with (a), stimulation delivery period is indicated by the blue bar that lasts 1.5 s. Conditions are the same as in Fig. 3.

To test the viability of the device for chronic IOS measurements, IOS responses were recorded from a mouse 40 days after implantation [Figs. 4(a) and 4(b)]. Interestingly, the response maps in general showed higher contrast under the same image processing conditions [SD (immediately after implantation): 47.2; SD (after 40 days): 57.9], whereby the outlines of the superficial vasculature were found to be more pronounced. This may be attributed to better postsurgical recovery from meningeal trauma after several weeks and the eventual clearance of the mixture of fluids (cerebrospinal fluid/blood emanating from dural vessels) that may have initially occluded the field of view.

### 3.2 Tissue Damage Evaluation via Immunohistochemical Analysis

**Fig. 5:**
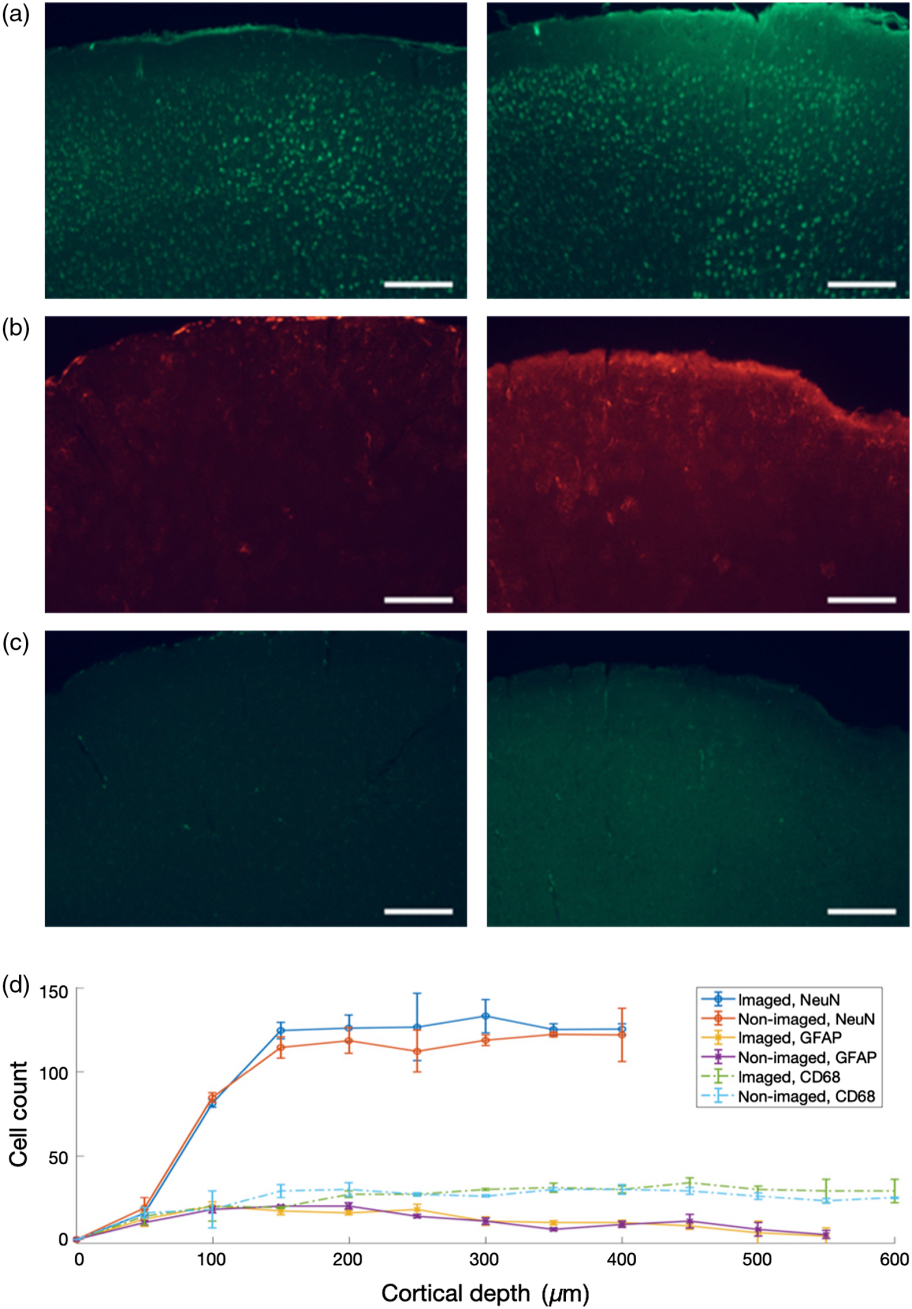
Immunostaining results stained with NeuN, GFAP, and CD68 antibodies for visualization of neuronal cell bodies, astrocytes, and microglia, respectively. (a)–(c) 40-μm coronal sections of the brain of a WT mouse used in an IOS recording session. Micrographs are representative of three mice (N=3) immunostained after IOS recordings under green and red illumination (30 min for each condition; total: 2 h). Left images show a section of the imaged area (irradiated with green and red LEDs for at least 2 h, cumulatively). Right images show corresponding sections in the opposite hemisphere that was not imaged. Scale bar: 200  μm. (d) Cell counts of the neurons (circles), astrocytes (crosses), and microglia (dots), indicating that the brain tissue did not exhibit appreciable immune response due to the device implantation and operation.

To evaluate whether that the cortical surface has maintained its integrity and no appreciable immune response occurred at the FOP-dura interface, we have performed immunohistochemical analysis of the brain tissue. Immunostaining of sliced brain tissue extracted immediately after the IOS recording session (∼2  h) showed that the distribution of glia (namely, astrocytes, stained with anti-GFAP; and microglia, stained with anti-CD68) in the imaged area was not significantly different to that of the opposite, nonimaged area: p<0.05 for both astrocytes (z score: −0.05774) and microglia (z score: −0.51962) in a two-tailed Mann-Whitney U test (Fig. 5). Moreover, neuron cell body distribution visualized using anti-NeuN was also not significantly different across hemispheres (p<0.05, z score: 1.41451, two-tailed Mann-Whitney U test). These results indicate that the implantation and operation of the device did not trigger an adverse immune response from glial cells and that the supragranular layers of cortical tissue did not undergo radiation- or heat-induced necrosis, confirming the viability of the imaging device in long and chronic recording sessions.

### 3.3 Cortical Surface Recordings in a Freely Moving Mouse

**Fig. 6:**
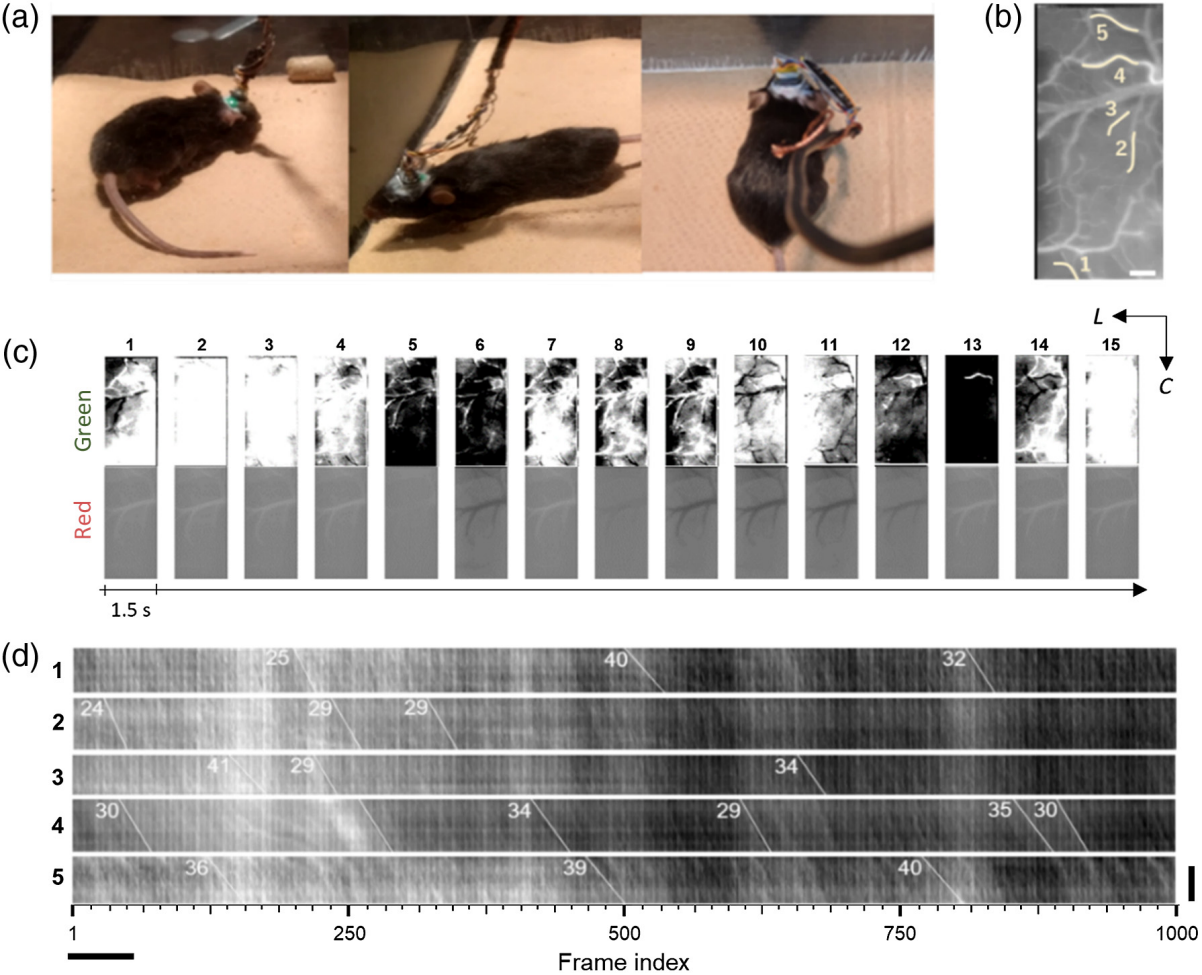
*In vivo* cortical imaging in a freely moving mouse. (a) Still images depicting an awake, freely behaving mouse prior to *in vivo* imaging session. The area of the enclosure was 30  cm×30  cm (Video 1, MP4, 4.75 MB [URL: https://doi.org/10.1117/1.JBO.27.2.026501.1]). (b) Brain surface image (averaged from 1000 frames) taken under green LED illumination by the device from the experiment. The beige lines indicate the five line ROIs used for the blood flow velocity estimation (Video 2, MP4, 2.10 MB [URL: https://doi.org/10.1117/1.JBO.27.2.026501.2]). Color bar matches the LUT under green illumination: $-2.3\times 10^{-3}$ to $1.5\times 10^{-3}$ $\Delta I/I_{0}$. Scale bar: 200  μm. (c) Activity maps constructed from 1.5-s bins of cortical recording from the freely moving mouse. LUT: $-2.3\times 10^{-3}$ to $1.5\times 10^{-3}$ $\Delta I/I_{0}$ (green); $-0.5\times 10^{-3}$ to $0.5\times 10^{-3}$ $\Delta I/I_{0}$ (red). (d) Results of the kymographic line scans of the five labeled ROIs traced along the blood vessels. Angles θ of the diagonal lines were used to estimate changes in the blood flow speed (ν=F·ΔP·tan θ). ν, blood flow speed; F, frame rate of the recording; ΔP, pixel pitch. The frame rate used was 132.82 fps and the 1000-frame recording lasted 7.52 s. Scale bar: 200  μm (vertical); 0.5 s (horizontal).

The imaging experiment on a freely moving mouse was performed as a proof-of-principle that the modular device is capable of recording IOS with a stable of field of view and without hampering the locomotion of the animal. Figure 6(a) shows still images of an awake mouse behaving naturalistically inside a 30  cm×30  cm acrylic enclosure. These images were taken prior to the imaging session, in which point the ambient light was turned off to minimize stray light that might degrade the imaging data. An average image of the cortical surface under green LED illumination is depicted in Fig. 6(b), showing well-defined contours of superficial blood vessels. As with Figs. 3 and 4, activity maps were constructed from 1.5-s bins of cortical recording from the freely moving mouse [Fig. 6(c)].

For the blood flow estimation, multiple θ values ranging from 25 deg to 41 deg (0.44 to 0.72 rad) were determined from perceptible striations across different time epochs for the five line ROIs, from which estimates of blood flow speed were obtained (Table 2). The minimum estimated cortical blood flow speed through this analysis was 0.47  mm/s (corresponding to 0.44 rad), and the maximum speed was 0.87  mm/s (0.72 rad). The mean frame rate of the recording was 132.82 fps, and the pixel pitch of the image sensor was 7.5  μm.

**Table 2:** Estimated cortical blood flow velocity in an awake, freely moving mouse obtained through kymographic analysis.

Angle (deg)	Flow velocity (mm/s)	Angle (deg)	Flow velocity (mm/s)
25	0.47	34	0.67
26	0.49	35	0.70
27	0.51	36	0.72
28	0.53	37	0.75
29	0.55	38	0.78
30	0.56	39	0.81
31	0.60	40	0.84
32	0.62	41	0.87
33	0.65		

## 4 Discussion

### 4.1 Intrinsic Optical Signals for Monitoring Cortical Dynamics

The distinct absorbance spectra of oxygenated hemoglobin (oxyhemoglobin, HbO) and deoxygenated hemoglobin (deoxyhemoglobin, HbR) enable us to monitor valuable physiologic processes that arise as a consequence of neurovascular coupling.^19^^,^^51^ Since the absorbance of oxygenated and deoxygenated hemoglobin reaches an isosbestic point around the wavelength range of the green LED ($\lambda_{\text{center}}=535\;\mathrm{nm}$), observing reflectance changes in the brain tissue using green LEDs as light sources gives rise to IOS that are strongly correlated with blood volume. On the other hand, in the orange-red ($\lambda_{\text{center}}=625\;\mathrm{nm}$) wavelength range, the absorbance of deoxyhemoglobin is larger than that of oxyhemoglobin such that using a light source in this wavelength range allows us to obtain IOS that are strongly correlated with oxygen concentration in the blood. Thus imaging under green illumination provides mainly volumetric information about hemodynamics, whereas red illumination provides oximetric information or the instantaneous oxygenation state within localized regions of the cortex.

Receptive fields in the primary somatosensory cortex (S1) are known to generate localized IOS in response to perturbations in their corresponding sensory units. In particular, within the HL receptive field of the S1, a robust characteristic triphasic profile can be observed immediately after mechanical stimulation of the contralateral HL (i.e., opposite to the hemisphere of the brain of the mouse). Various configurations of the HL mechanical stimulation procedure have been realized, ranging from constant cutaneous press to vibratory stimulation using high-frequency piezoactuators^6^ at various illumination wavelengths,^52^^,^^53^ giving rise to IOS profiles of slightly varying temporal dynamics.^20^^,^^46^^,^^51^ In this study, the S1HL of anesthetized mouse was stimulated via a constant press to the plantar part of the hindpaw in both contralateral and ipsilateral configurations. Urethane was used because it permits extended periods of imaging when administered as systemic anesthesia. Furthermore, for sensory stimulation investigations, using urethane is advantageous as neural responses remain relatively unattenuated and were found to be less irregular across trials compared to other anesthetics such as isoflurane.^16^

Further developments on the IOS imaging modality in freely moving studies are also expected to have profound implications on the interpretation of functional magnetic resonance imaging data since the latter, a popular whole brain imaging method that has already found widespread clinical application, is also known to rely on oxy- and deoxyhemoglobin conversion to report functional activity.^13^^,^^51^^,^^54^^,^^55^

### 4.2 Practical Considerations of IOS Recording

In order to gain a more accurate understanding of how the nervous system processes sensation and perception, particularly in the cerebral cortex, it is essential to develop tools that can probe the intact brain in living subjects with as minimal interference as possible.^23^^,^^29^^,^^54^^,^^55^ One promising strategy is to use optical imaging to monitor changes in the optical properties of individual cells, tissue, or blood vessels as the animal performs a specific task or receives sensory stimuli.^48^^,^^56^^,^^57^ Various somatosensory, motor, and cognitive functions relating to the topography and connectivity within the cortex have already been identified, including certain aspects of sensorimotor organization that are integral to active sensing,^11^ features of sensory processing that enable selective attention,^58^ and pathophysiological characteristics of neuroplasticity that contribute to chronic pain.^59^ However, studying the multitudinous functional states within the cortex of small animals exhibiting naturalistic behavior remains difficult to implement and can be considered an open challenge that could be addressed using contact imaging approaches.

Here we have presented a modular, CMOS-based, head-mounted cortical imaging device that was developed for monitoring IOS and related hemodynamic activity in wild-type mice. The ability to monitor functional changes in cortical activity in the absence of extrinsic fluorescent sensors (e.g., BAPTA-based dyes) or genetically modified reporters (e.g., GCaMP and other GECIs) eliminates the risk of the molecular probes interfering with normal physiological processes which may render some findings invalid. Simplifications in the imaging scheme is possible since emission filters,^60^ which are ubiquitous in fluorescence imaging modalities, are typically unnecessary for IOS imaging^13^^,^^17^ albeit the signal-to-noise ratio of the latter is markedly lower.^52^^,^^53^^,^^61^ In spite of this, IOS imaging offers adequate spatial and temporal resolution for visualizing the boundaries of receptive fields, which are useful in constructing topographic maps.^62^

The current modular design of the device is a result of several iterations that were aimed at improving the mounting stability on the head of the mouse, ease of handling, packing LEDs of different center wavelengths for IOS imaging, and keeping the device as light as possible. Figure S4 in the Supplementary Material shows the evolution of the frames that were used in *in vivo* imaging experiments. Such changes have afforded a more standardized imaging setup and enabled a higher experiment turnout. The new design also made the device more accessible to the community as it allows practitioners with moderate technical skill to carry out their own IOS imaging experiments. Furthermore, in addressing some of the limitations of the prototype described previously,^41^ two-color illumination was introduced in this study and a lightweight, compact relay board was developed for better noise suppression.

Through the use of LEDs, which are highly energy-efficient light sources, heat generation was expected to be minimal during operation.^63^ In addition, because the LEDs were embedded on the sensor plate component of the device and therefore not in direct contact with the mouse, they posed even less risk in increasing the temperature of the cortex outside a safe range. Regarding the stability of the LED illumination between IOS imaging sessions (days 1 and 40), we have standardized the forward operating voltage of the LED arrays to be 2.74 V (green) and 1.63 V (red), whereas the forward current values were 7 mA (green) and 2 mA (red). Light power obtained from the LED arrays was found to remain at 8.1 and $1.5\;\mathrm{mW}/{\mathrm{mm}}^{2}$ for green and red illuminations, respectively. A limitation of the current system persists, however, in that the absence of a direct line-of-sight (as in an epifluorescence setting) between the LED sources and the imaged area of the tissue necessitates a fraction of the illumination to traverse the bulk of the surrounding (nonimaged) tissue before being detected by the image sensor. Changes in the absorption properties of this surrounding tissue during cortical functional activation may influence the resulting response maps, the extent of which has not been characterized in the study.

Since vascular features can be imaged at sufficiently high frame rates (133 fps) under green illumination, a kymographic analysis for estimating blood flow speed in superficial blood vessels was also demonstrated. This additional hemodynamic parameter can be used to complement IOS analysis. Differential imaging against a background frame has allowed detection and tracking of erythrocytes within the vessels, which formed the basis of the estimation procedure for blood flow speed.^64^^,^^65^ Kymographs were constructed from line ROIs overlaid along the central lumen of representative blood vessels. Striations in the kymographs arise from the heterogeneous composition of blood, where dark bands are attributed to a higher local concentration of erythrocytes.^65^ Furthermore, the incline corresponds to the motion of the erythrocytes across time (i.e., from one frame to the next). However, because the striations in the kymographs were relatively thick compared to the pixel pitch as a direct result of the dark bands of the erythrocytes spreading over a large area within the blood vessels in the recordings, θ measurements were limited to only two significant figures. Increasing the frame rate may be able to partially remedy this as the temporal axis would scale up, therefore improving sensitivity in terms of increasing the number of angles that can practically be differentiated from each other, given the same time interval.

A more extensive analysis of the intrinsic signal profiles in response to HL tactile stimulation and measurement of the relative contribution between volumetric and oximetric changes to IOS response are outside the scope of the current report and will be discussed elsewhere. In the present investigation, imaging experiments were mostly carried out in anesthetized preparations due to the ease with which stimuli can be precisely and consistently delivered to a body part of a sedated mouse. IOS signals time-locked to a predefined trigger could not be retrieved for the freely moving session. However, differential activity could still be observed in the somatosensory cortex of an active mouse for both green and red LED illuminations. The imaging area also did not exhibit discernible distortions during the freely moving session, suggesting that the device was firmly attached to the head of the mouse. In such a case, the length of the recording can be extended and configured to accommodate a variety of behavioral paradigms, conditioning schedules, or maze tasks.^66^^–^^68^ Although interference and noise cannot be completely removed in the transmission of analog signals, installation of a high-grade twisted wire cable with an appropriate shielding scheme and incorporation of the relay board were measures that we have taken to reduce noise in the output images.

Constructing a stimulation protocol that will deliver spatially precise and reproducible tactile stimulus on the HL of an active, unrestrained mouse is being developed for future studies to make full use of the lightweight cortical imaging device. Nevertheless, in its current state, the small form factor and light weight of the device has already been shown to facilitate mouse experimental studies in freely moving conditions.

## 5 Conclusions

In this study, we demonstrated long-term observation of IOS in mice using a modular head-mounted imaging device. The lightweight device incorporates a CMOS image sensor that is mounted on a PCB embedded with green and red LEDs for monitoring blood volumetric and oximetric changes, respectively. Aluminum jigs incorporated with FOPs were designed to simplify the assembly of the sensor plate and head plate components of the device. By taking the approach of directly imaging the specimen surface (contact imaging) without the use of lenses, miniaturization was made possible while maintaining adequate spatial and temporal resolutions.

We observed IOS in the HL receptive field of the somatosensory cortex in response to mechanical stimulation (1.5 s) of anesthetized mice. Trial averaging was used to construct response maps of the cortex, revealing differential activity between contra- and ipsilateral stimulation. Contralateral IOS recordings exhibited characteristic profiles during green and red LED illumination and were time-locked to the onset of contralateral stimulation. Ipsilateral IOS recordings showed weaker initial response but also generated regular late-occurring peaks. Although current experiments have focused on validating the technique by obtaining stereotypical cortical responses from standard stimulation procedures in anesthetized subjects, this imaging approach readily lends itself to neuroscientific studies in freely moving conditions and can also support a range of imaging modalities (e.g., fluorescence, IOS, and vascular dynamics/flow velocity). Through our imaging platform, we anticipate that certain relationships between these optical phenomena and cortical dynamics may be identified which could, in turn, aid in developing better diagnostic approaches for treating neurological disorders.
