# Supplementary material for: Modular head-mounted cortical imaging device for chronic monitoring of intrinsic signals in mice
Source: J Biomed Opt. 2022 Feb 14;27(2):026501. doi: 10.1117/1.JBO.27.2.026501 (PMC8843356; doi:10.1117/1.JBO.27.2.026501)
Supplement: Supplementary file 1 [file JBO_027_026501_SD001.pdf]

Supplementary information

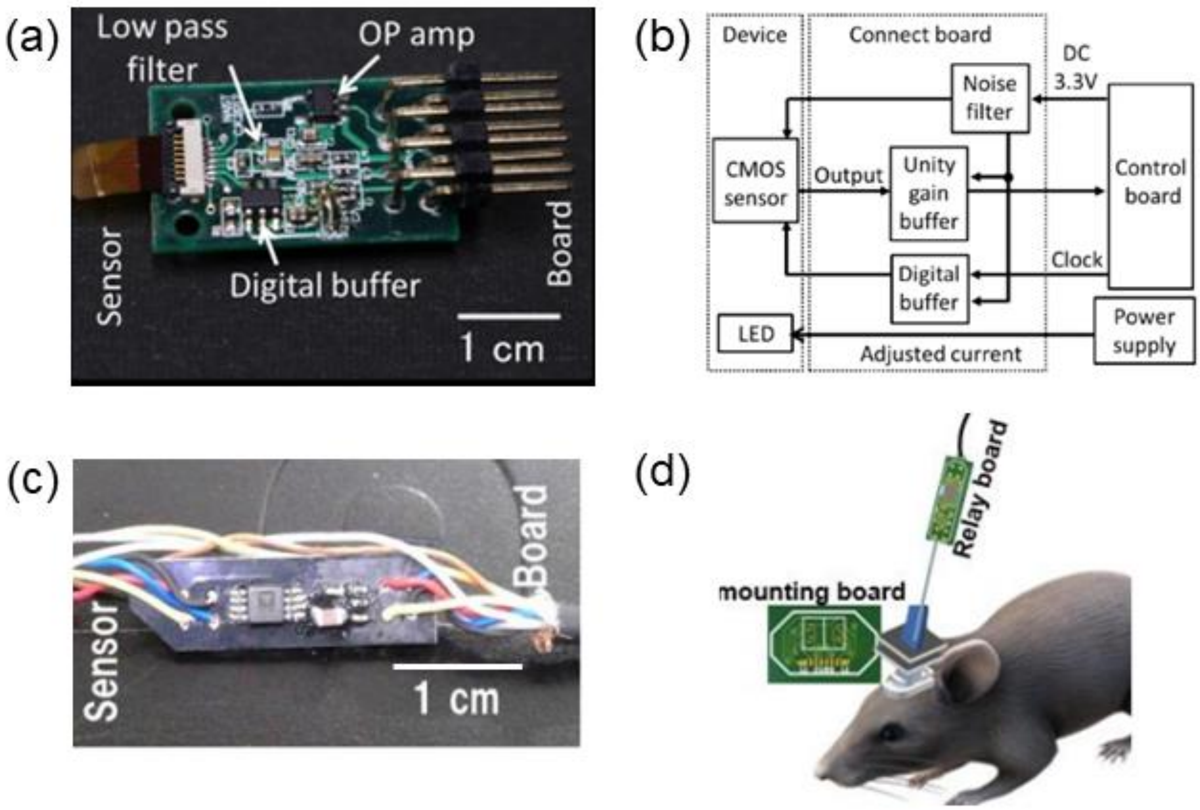

Fig. S1 Miniaturization of the relay board. (a) Previous design of the relay board [39], showing the low pass (noise) filter, operational amplifier and digital buffer. The relatively bulky circuit board was secured to the back of the mouse or rat. (b) Circuit diagram for (a). (c) Current design of the relay board, sporting similar functionality. (d) Schematic of the mouse mounted with the modular device. The relay board is sufficiently light to hover above the mouse during *in vivo* freely moving experiments. The surface of the mounting board of the sensor plate is also shown.

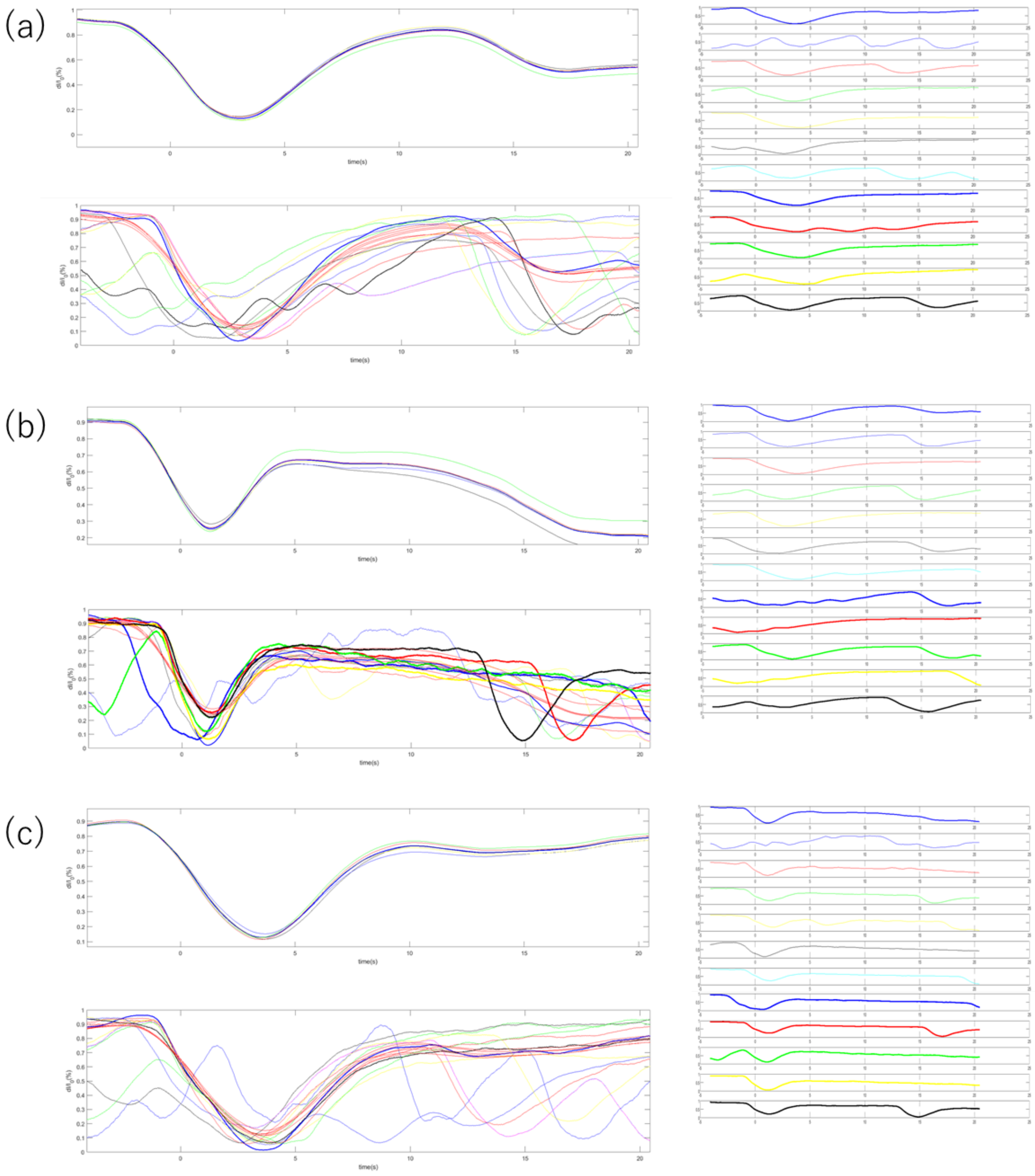

Fig. S2 Contralateral intrinsic optical signal (IOS) responses in the hindlimb receptive field (S1HL) under green illumination (λ_{center}= 535 nm) in three anesthetized wild-type mice. These line plots were constructed prior to the detrending step. (a-c) Line plots of the fractional change in intensity values ($\Delta I/I_0$) within the cortical recordings. The interval from 0 to 1.5 s is the stimulation delivery period. *Top left panels:* $\Delta I/I_0$ line plots from five regions of interest (ROIs, 20 px × 20 px) within the cortical recordings and averaged across 11 trials. These trials are representative of all 20 trials. *Bottom left panels:* Individual $\Delta I/I_0$ line plots from the first 11 trials of the set superimposed on each other. The thick dark blue line is the average plot. *Right panels*: Same 11 trials in bottom left panels but plotted individually. Note the slightly different x-axis range. Second dips of comparable magnitude to the first dip are apparent at around 15 s in multiple line plots.

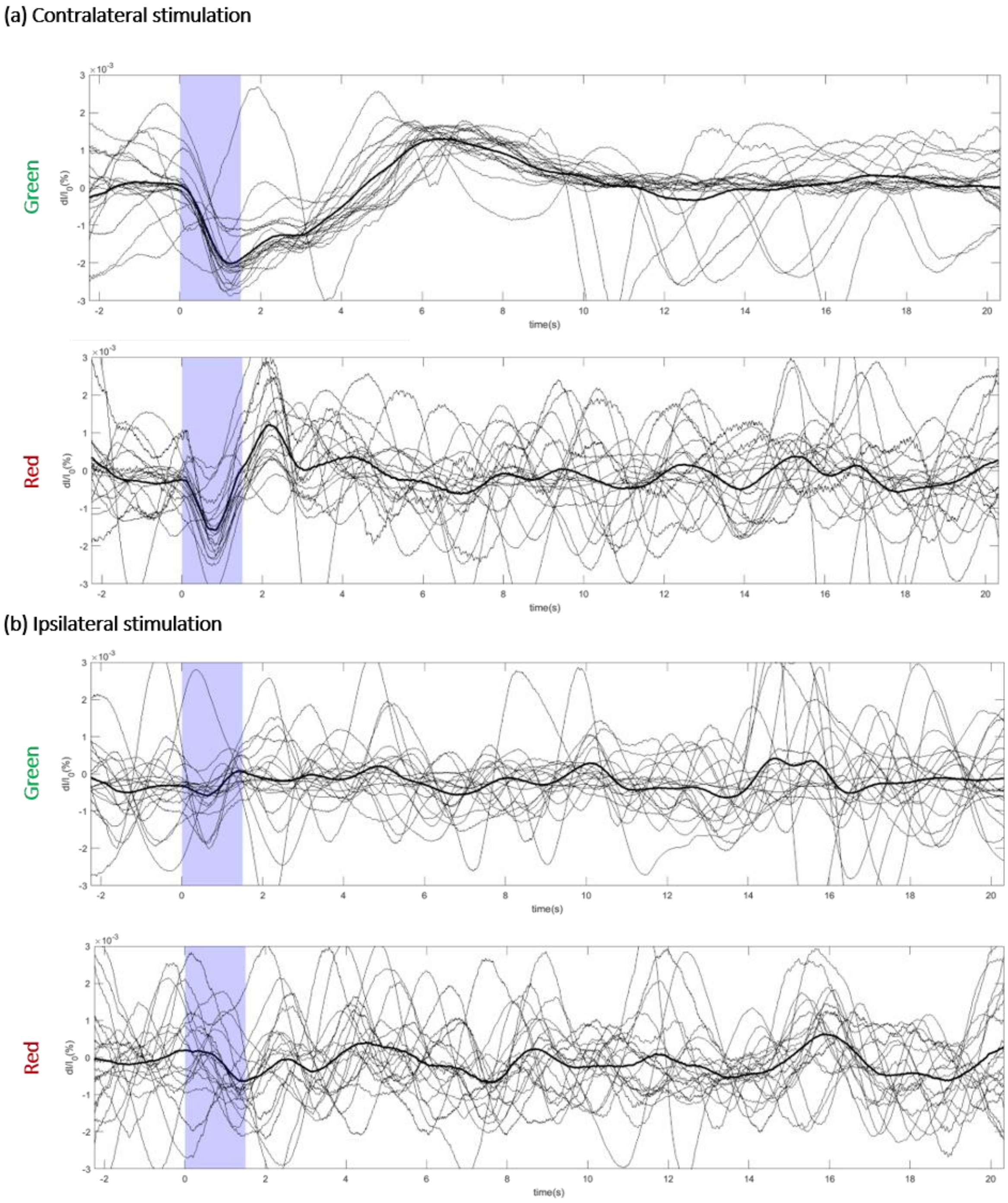

Fig. S3 Contralateral (a) and ipsilateral (b) intrinsic optical signal (IOS) responses in the hindlimb receptive field (S1HL) of an anesthetized wild-type mouse. Line plots of the fractional change in intensity values ($\Delta I/I_0$) of the IOS for individual trials (regular lines) and their mean (thick line), shown under green (*Top panels*; λ_{center}= 535 nm) and red illumination (*Bottom panels*; λ_{center}= 625 nm) The interval from 0 to 1.5 s is the stimulation delivery period. Note the time-locked IOS responses upon contralateral stimulation.

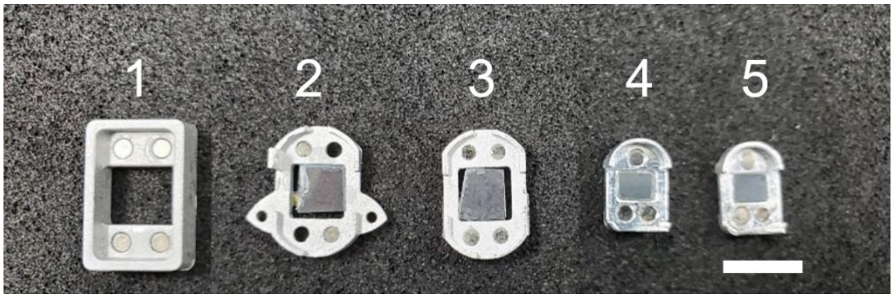

Fig. S4 Design of headplate jig frames used in *in vivo* experiments. The frame labeled 1 is oldest while 5 is newest. Scale bar: 1 mm.
